# Toward understanding the molecular mechanism of a geminivirus C4 protein

**DOI:** 10.1080/15592324.2015.1109758

**Published:** 2015-10-22

**Authors:** C Michael Deom, Katherine Mills-Lujan

**Affiliations:** Department of Plant Pathology; University of Georgia; Athens, GA USA

**Keywords:** AtSK, BCTV, C4 protein, curtovirus, cell cycle, geminivirus, hyperplasia, replication

## Abstract

Geminiviruses are ssDNA plant viruses that cause significant agricultural losses worldwide. The viruses do not encode a polymerase protein and must reprogram differentiated host cells to re-enter the S-phase of the cell cycle for the virus to gain access to the host-replication machinery for propagation. To date, 3 *Beet curly top virus* (BCTV) encoded proteins have been shown to restore DNA replication competency: the replication-initiator protein (Rep), the C2 protein, and the C4 protein. Ectopic expression of the BCTV C4 protein leads to a severe developmental phenotype characterized by extensive hyperplasia. We recently demonstrated that C4 interacts with 7 of the 10 members of the *Arabidopsis thaliana* SHAGGY-like protein kinase gene family and characterized the interactions of C4 and C4 mutants with AtSKs. Herein, we propose a model of how C4 functions.

Geminiviruses (family *Geminiviridae*) are small plant DNA viruses with circular single-stranded genomes that cause significant losses in food, fiber, and cash crops worldwide.^1^ The family consists of 7 genera: *Becurtovirus, Curtovirus, Eragrovirus, Mastrevirus, Topocuvirus*, and *Turncurtovirus* have monopartite genomes, while the *Begomovirus* can have mono- or bipartite genomes.^2^ The absence of a polymerase gene in geminiviruses requires that the S-phase of the cell cycle be reactivated in terminally differentiated cells following virus infection to gain access to cellular DNA replication machinery for virus propagation. The replication-initiator protein (Rep) is present in all geminiviruses and is the only viral protein essential for replication. It is required to restore DNA replication competency to terminally differentiated host cells.^3,4^ In addition, in the curtovirus genus, the *Beet curly top virus* (BCTV) C2 protein is involved in enhancing replication competency, although the molecular mechanism is unclear,^5^ and the BCTV C4 protein has been shown to restore DNA replication competency.^6^

Most geminiviruses do not induce cell proliferation, but some curtovirus C4 proteins are very proficient at regulating cell cycle progression and promoting mitosis.^6-9^ The BCTV C4 protein induces vein swelling and enations in BCTV-infected hosts and hyperplasia in transgenic *Arabidopsis* plants.^6-10^ Curtovirus *C4* genes, like all geminivirus *C4* and *AC4* genes (the *C4* gene in bipartite begomoviruses is designated *AC4*), are nested within the *Rep* genes and encode small proteins of approximately 10 kDa, depending on the virus. The BCTV C4 protein interacts with members of the *Arabidopsis* SHAGGY-like protein kinase (AtSK) family.^7,11,12^ AtSKs are homologues of the glycogen synthase kinase 3 (GSK3) family of serine/threonine kinases (GSK3α and GSK3β) in animals.^13^ However, in plants the AtSK gene family has evolved into 10 members to address diverse plant-specific functions.^14,15^ Seven AtSK members have been implicated in brassinosteroid (BR) signaling.^16^ The BRs are steroid hormones that regulate plant growth and development. We and others showed that expression of C4 in transgenic *Arabidopsis* disrupts the BR pathway, indicating a direct role for C4 in regulating BR signaling.^7,11,12^

## C4 protein from Beet curly top virus interacts with multiple AtSKs

We recently showed that C4 interacts with 7 of the 10 AtSKs (clade 1 AtSKs-11,-12,-13; clade 2 AtSKs-21,-22,-23; and clade 3 AtSK32),^12^ the same 7 AtSKs involved in regulating BR signaling (**Table 1**). Bikinin, a selective inhibitor of the 7 AtSKs implicated in BR signaling, induced hyperplasia in *Arabidopsis* similar to that induced by C4 (**Table 1**).^12^ An array of functions are attributed to AtSKs,^16^ especially AtSK21, which is the most well studied AtSK. The numerous cellular functions regulated by AtSK21 suggests that other AtSK family members may also regulate a wide variety of overlapping and individual AtSK-specific functions.^15,16,17^ Even within redundant functions, the degree of substrate specificity can vary between AtSKs.^18^ In some instances, preferential tissue-specific AtSK expression is observed,^15^ and it is likely that expression levels of individual AtSKs vary at different stages of development. Therefore, C4 likely impacts a large number of diverse functions regulated by clade 1 AtSKs, clade 2 AtSKs and AtSK32.

**Table 1.** *Arabidopsis thaliana* SHAGGY-like protein kinase family.

Clade	Name	Locus Identifier	C4 Interaction/BR Signaling/Bikinin Inhibition
1	AtSK11	AT5G26751	+
	AtSK12	AT3G05840	+
	AtSK13	AT5G14640	+
2	AtSK21	AT4G18710	+
	AtSK22	AT1G06390	+
	AtSK23	AT2G30980	+
3	AtSK31	AT3G61140	−
	AtSK32	AT4G00720	+
4	AtSK41	AT1G09840	−
	AtSK42	AT1G57870	−

## Requirements for a functional C4/AtSK interaction

The C4/AtSK interactions and C4 function require the presence of a phosphorylated Ser/Thr residue at amino acid position 49.^12^ In addition, AtSKs must be catalytically active to interact with C4, supporting their role in phosphorylating C4.^12^ Residue 49 is part of a proline-directed Ser/Thr kinase phosphorylation motif (Ser/Thr-Pro). Ser/Thr-Pro motifs are phosphorylated by members of a large family of proline-directed Ser/Thr kinases in eukaryotes, including AtSKs, and regulate a diverse array of cellular processes.^19^ While most peptide bonds have the more energetically favored *trans* isomer, proline can exist in either a *cis* or *trans* conformation. Phosphorylation of Ser/Thr-Pro motifs limits the rate of *cis/trans* isomerization, which plays an important role in regulating protein structure,^19^ and may be essential in regulating C4 function.

## C4 requires plasma membrane localization for function

Geminivirus C4/AC4 proteins contain a conserved N-myristoylation motif required for localization to the plasma membrane (PM).^11,20^ We recently showed that BCTV C4/AtSK complexes localize primarily to the PM and nucleus. Disruption of the C4 N-myristoylation motif resulted in a nonfunctional C4 mutant, C4G2A, that failed to induce hyperplasia. The mutant retained the ability to bind AtSKs, but localized to the cytosol and nucleus, indicating that the formation of C4/AtSK complexes does not require association with the PM, but that functional C4/AtSK complexes do require PM localization.^12^ The targeting of a portion of both C4G2A/AtSK and C4/AtSK complexes to the nucleus suggests that nuclear localization is not necessary to induce hyperplasia. Similarly, the AC4 protein of the *East African cassava mosaic Cameroon virus* (EACMCV) required an N-myristoylation motif and PM localization for function. Interestingly, the AC4 protein suppresses the systemic phase of RNA silencing and has not been shown to induce hyperplasia.^20^

N-myristoylation alone is likely not sufficient to stably anchor a protein to the PM.^21,22^ A second signal in the myristoylated protein, such as another fatty acyl chain, a polybasic group of amino acids that binds the negatively charged phospholipids within the PM, or a domain that interacts with another membrane protein is required for stability. Indeed, PM localization of the AC4 protein of EACMCV was shown to require palmitoylation of Cys 3 in addition to myristoylation of Gly 2 in the *N*-myristoylation motif.^20^ While BCTV C4 requires Gly2 for myristoylation,^11^ a second signal has not been identified. Two likely possibilities are the sole Cys9 residue in C4, which is a putative site for palmitoylation, and/or the 3 basic amino acids Lys13, Lys15, and Arg17. The latter would be similar to the Src protein of *Rous sarcoma virus*, where 3 basic amino acids at the N-terminus of the protein are necessary to stabilize localization of the myristoylated protein to the PM.^23^ Indeed, a mutation in C4 of Lys13 to Ala13 resulted in a very mild C4-like phenotype.^12^

## How does the BCTV C4 protein induce hyperplasia?

**Figure 1.**
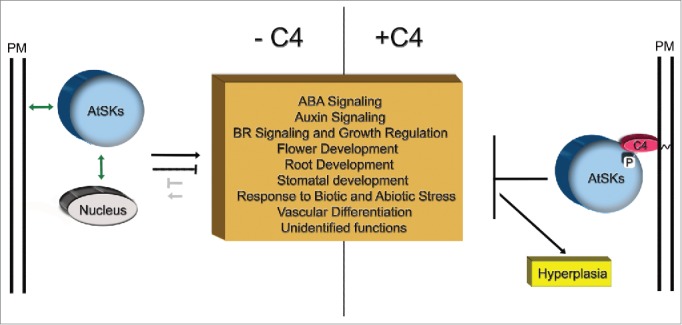
C4/AtSK interaction model. In the absence of C4, AtSKs (clade 1 AtSKs, clade 2 AtSKs and AtSK32) activate (black arrow) or inhibit (black bar) proteins involved in the various cellular processes indicated (brown box). In a few instances, cellular proteins activate (gray arrow) or inhibit (gray bar) AtSKs. In the absence of C4, AtSKs may be localized to the cytosol, nucleus and/or PM. Double-head arrows indicate AtSKs association/disassociation with the nucleus and PM. In the presence of C4, AtSK activities are inhibited (black bar). Detailed description of activation or inhibitory functions attributed to specific members of clade 1 and clade2 AtSKs, as well as AtSK32 and cellular proteins are described.^16^.

While extensive details of the mechanism underlying the function of the C4 protein remain unknown, our recent findings,^12^ and previously published work,^7,11^ provide a basis to propose a tentative mechanistic model of how the BCTV C4 protein functions (**Fig. 1**). As indicated above, the ability of the C4 protein to restore replicational competency, as manifested by extensive hyperplasia, requires the formation of functional C4/AtSK complexes at the PM. Therefore, inhibition or modulation of AtSK function(s) by C4 likely occurs at the PM. This is supported by the fact that the presence or absence of C4 influences specific AtSK-PM interactions. In the absence of C4, clade 2 AtSKs localized to the cytosol, nucleus, and punctate regions on the PM.^12^ This is in line with previous reports of AtSKs localizing to the PM; AtSK21 was localized to the nucleus, cytosol, and PM and AtSK41 was localized to the cytosol and PM.^24-26^ When C4 is present, C4/clade 2 AtSK complexes are distributed evenly along the PM membrane. This shift in localization is C4-dependent, since the PM localization of AtSK41, which does not interact with C4, is unchanged in the presence of C4.^12^ More importantly, recent evidence suggests that PM localization is critical for some AtSK function, AtSKs (putatively clade 1 and clade2) were shown to interact with the tracheary element differentiation inhibitory factor receptor (TDR) at the PM and play a crucial role in regulating xylem cell differentiation.^26^ Furthermore, in *Drosophila*, a PM-proximal GSK-3 activates the Wnt signaling pathway by phosphorylating LRP6, a transmembrane protein, that is critical for signal transduction.^27,28^ Similarly, the bikinin-induced hyperplastic phenotype could be explained by bikinin inhibiting the function of AtSKs that function at the PM.

While current experimental data support the model presented, additional information is needed to elucidate details on how the C4 protein modulates the host cell cycle. Future experiments to identify specific C4/AtSK complexes involved in the induction of hyperplasia, possible additional C4-interacting host proteins, and possible host proteins that interact with C4/AtSK complexes or modified C4/AtSK complexes will provide additional insights into how the BCTV C4 protein usurps the host cell cycle to promote mitosis.
